# Preventive Effects of a Natural Anti-Inflammatory Agent, Astragaloside IV, on Ischemic Acute Kidney Injury in Rats

**DOI:** 10.1155/2013/284025

**Published:** 2013-06-19

**Authors:** Shufeng Tan, Guofu Wang, Yongping Guo, Dingkun Gui, Niansong Wang

**Affiliations:** ^1^Health Examination Center, Sixth People's Hospital Affiliated to Shanghai Jiao Tong University, Shanghai 200233, China; ^2^Zhejiang Provincial Key Lab of Geriatrics, Zhejiang Hospital, Hangzhou 310013, China; ^3^Department of Nephrology and Rheumatology, Sixth People's Hospital Affiliated to Shanghai Jiao Tong University, Shanghai 200233, China

## Abstract

This study investigated the anti-inflammatory effects of astragaloside IV(AS-IV) on ischemia/reperfusion (IR) induced acute kidney injury (AKI) in rats. Experimental model of ischemic AKI was induced in rats by bilateral renal artery clamp for 45 min followed by reperfusion of 12 h and 24 h, respectively. AS-IV was orally administered once a day to rats at 10 and 20 mg·kg^−1^·d^−1^ for 7 days prior to ischemia. AS-IV pretreatment significantly decreased serum urea, creatinine, and cystatin C levels at 12 h and 24 h of reperfusion in AKI rats. AS-IV pretreatment also ameliorated tubular damage and suppressed the phosphorylation of p65 subunit of NF-**κ**B in AKI rats. Moreover, NF-**κ**B and MPO activity as well as serum and tissue levels of TNF-**α**, MCP-1, and ICAM-1 were elevated in AKI rats. All of these abnormalities were prevented by AS-IV. Furthermore, AS-IV downregulated the mRNA expression of NF-**κ**B, TNF-**α**, MCP-1, and ICAM-1 in AKI rats. These results suggest that AS-IV might be developed as a novel therapeutic approach to prevent ischemic AKI through inhibition of NF-**κ**B mediated inflammatory genes expression.

## 1. Introduction

Acute kidney injury (AKI) is common in intensive care unit (ICU) and is independently associated with high mortality and morbidity [1–3]. Renal ischemia-reperfusion (I/R) injury is an important cause of AKI as observed after renal transplantation, cardiac and vascular surgery, septic as well as hypovolemic shock, and trauma [4]. Unfortunately, innovative interventions beyond supportive therapy are currently not available for ischemic AKI. Thus, there is an urgent need for the development of novel and effective therapeutic approaches to prevent ischemic AKI. Inflammatory response is now believed to play a central role in the pathophysiology of ischemic AKI. IR-induced inflammatory responses, including excessive generation of cytokines, overexpression of surface adhesion molecules, and chemotactic proteins, make the inflammatory cells extravasculate from the blood stream and attract to the kidney tissues [5, 6]. The vascular endothelium plays a crucial role in the initiation of inflammatory responses [7]. Inflammatory cascades that are initiated by endothelial dysfunction can be augmented by the generation of many inflammatory mediators, including proinflammatory cytokines and chemotactic cytokines, by the ischemic proximal tubule [8–12]. Increased plasma levels of proinflammatory cytokine predict mortality in patients with AKI [12]. Adhesion molecules are required for leukocyte adhesion during inflammatory processes. Leukocyte adhesion to endothelial cells induces inflammation and extension of cellular injury [13]. Numerous studies have demonstrated that ICAM-1 plays an important role in the pathophysiology of AKI [14, 15]. Therefore, inflammation is now believed to have a central role in the pathogenesis of ischemic AKI.

NF-*κ*B, a family of transcription factors, regulates the gene expression of several cytokines and chemotactic proteins involved in renal inflammation [16]. There is mounting evidence indicating a critical role of NF-*κ*B in the pathogenesis of renal IR injury, which is a major cause of AKI. Activation of NF-*κ*B has a major role in the pathophysiology of experimental ischemic AKI [17]. There is more specific evidence linking NF-*κ*B with ischemia AKI. Previous experimental study has reported that in vivo transfection of NF-*κ*B decoy oligodeoxynucleotides inhibits renal injury, leukocytic infiltration, and inflammatory mediators in ischemic AKI [18]. These studies strongly suggest that NF-*κ*B-mediated inflammatory processes represent an important mechanism leading to AKI. Therefore, anti-inflammatory strategies that targeting inhibition of NF-*κ*B pathway may effectively prevent ischemic AKI. 

Astragaloside IV(AS-IV) is one of the major and active components of the Astragalus membranaceus (Fisch) Bge. The chemical structure of astragaloside IV (C_41_H_68_O_14_, molecular weight = 784) was described in our previous study [19]. It was reported that AS-IV ameliorated IR injury in various organs, including heart [20] and brain [21]. In vitro study has demonstrated the anti-inflammatory effects of AS-IV [22]. Recent experimental studies have reported the anti-inflammatory activity of AS-IV in a murine model of chronic asthma [23] and in rats with focal cerebral IR injury [24]. However, the protective effects of AS-IV on inflammatory processes in ischemic AKI have not been investigated yet. The purpose of this study is to test the hypothesis that AS-IV prevents inflammation in ischemic AKI by suppressing NF-*κ*B mediated inflammatory genes expression and then provide new insights into the field of ischemic AKI therapy.

## 2. Materials and Methods

### 2.1. Drug Preparation

Astragaloside IV(AS-IV) was purchased from Xi'an Sobeo Pharmaceutical Technology Company, Limited (purity above 98%, Xi'an, China). AS-IV was suspended in 1% carboxymethyl cellulose (CMC) solution as a vehicle for its administration and was administered once a day to rats by oral gavage. The dosage of AS-IV used in this study was chosen as described in previous study [21].

### 2.2. Experimental Design and Animal Model

This study was approved by the Animal Ethics Committee of Fudan University, Shanghai, China. All animal procedures were performed in accordance with the “Guide for the Care and Use of Laboratory Animals” published by the National Institutes of Health. Seven-week-old male Sprague-Dawley rats, weighing 200 to 220 g, were purchased from Experimental Animal Center, Fudan University, Shanghai, China. Two experimental sets were designed according to the duration of 12 h or 24 h reperfusion. In each set, animals were further subdivided into the following groups (*n* = 8 per subgroup): (1) sham-operated rats pretreated with normal saline (Sham), (2) IR rats pretreated with CMC vehicle alone served as control (Veh), (3) IR rats pretreated with AS-IV at dose of 10 mg/kg (AL), and (4) IR rats pretreated with AS-IV at dose of 20 mg/kg (AH). AS-IV was orally administered once a day to rats for 7 days prior to ischemia. Rats were then anesthetized with pentobarbital sodium (50 mg/kg ip) and placed on a homeothermic table to maintain core body temperature at 37°C. Experimental model of ischemic AKI was induced in rats by bilateral clamping of the renal arteries for 45 min, followed by reperfusion for 12 h and 24 h, respectively. Sham-operated control rats underwent identical procedure but without bilateral renal artery clamping. At 12 h or 24 h of reperfusion, the rats were deeply anesthetized with pentobarbital (50 mg/kg), and the blood samples were taken from the abdominal aorta for determination of blood urea nitrogen (BUN), serum creatinine, cystatin C, and inflammatory mediators. The kidneys were then removed and bisected in the equatorial plane; the left kidney was snap-frozen in liquid nitrogen and stored at −70°C for protein and total RNA extraction, and the right kidney was fixed in buffered 10% formalin and prepared for routine histologic examinations. In an additional experiment, rats were deeply anesthetized with pentobarbital (50 mg/kg) at 4 h of reperfusion, and the left kidneys were removed for protein extraction. 

### 2.3. Renal Function and Histological Evaluation

Plasma levels of creatinine and BUN were determined by using an automatic biochemistry analyzer (Hitachi Model 7600 Series Automatic Analyzer, Japan). Cystatin C was a novel biomarker for early detection of kidney tubular injury. Thus, the serum cystatin C levels were determined using commercially available kit (The Guangdong Hongye Antibodies Technology Co., Ltd, China) in accordance with the manufacturer's instructions. The kidneys, fixed in a 10% neutral buffered formalin solution, were embedded in paraffin, cut into 4 *μ*m sections and then stained with hematoxylin and eosin. The sections were then examined by light microscopy. Histopathologic scoring was assessed by grading tubular necrosis, loss of brush border, cast formation, and tubular dilatation in 10 randomly chosen, nonoverlapping fields. The degree of kidney damage was estimated by the following criteria: 0, none; 1, ≤10%; 2, 11–25%; 3, 26–45%; 4, 46–75%; and 5, ≥76% [25]. The morphologic assessment was performed by the renal pathologist without knowledge of treatment. 

### 2.4. Determination of MPO Activity in Renal Tissues

The kidney myeloperoxidase (MPO) activity was used as a marker of renal neutrophil infiltration and activation. The MPO activity in renal tissue was determined using the commercial assay kits (Nanjing Jiancheng Bioengineering Institute, Nanjing, China), according to the manufacturer's instruction.

### 2.5. NF-*κ*B Activity Measurement

Nuclear extracts of kidney from the above-mentioned groups were prepared using the nuclear extract kit (Active Motif). Twenty micrograms of nuclear extract were used for the determination of NF-*κ*B activity with the TransAM NF-*κ*B p65 transcription factor assay kit (Active Motif) according to the manufacturer's instruction.

### 2.6. ELISA for Levels of Inflammatory Mediators in Serum and Renal Tissues

The serum and renal tissue levels of MCP-1 and ICAM-1 (Uscn Life Science Inc., Wuhan, China), as well as TNF-*α* (NeoBioscience Technology Company, Beijing, China) were determined using commercial enzyme-linked immunosorbent assay (ELISA) kits according to the manufacturer's instruction. For the measurement of TNF-*α*, MCP-1, and ICAM-1 levels in kidney tissues, a portion of each kidney was homogenized in phosphate-buffered saline containing 0.05% Tween 20. The samples were centrifuged at 3000 rpm for 15 min, and supernatant total protein concentration was quantitated. The supernatants were then stored at −70°C until the ELISA assays could be performed. The TNF-*α*, MCP-1, and ICAM-1 levels in kidney tissues were normalized to the protein content.

### 2.7. Western Blotting

Tissue protein was separated by sodium dodecyl sulfate (SDS)/polyacrylamide gel electrophoresis and transferred to a polyvinylidene difluoride membrane. The membranes were then blocked by incubation in Tris-buffered saline Tween (TBS and 0.1% Tween 20) containing 5% bovine serum albumin and incubated overnight at 4°C with p-NF-*κ*B p65 (Santa Cruz Biotechnology, USA) and NF-*κ*B p65 (Cell Signaling Technology, USA). Negative controls were performed without primary antibody. After washing, the horseradish peroxidase-labeled secondary antibody was added and incubated 1 h at room temperature. Lamin A (Santa Cruz Biotechnology, USA) was used as a loading control. Chemiluminescence detection was performed with the KC detection kit (KC-420, KangChen Biotechnology, Shanghai). Relative protein expression was described as the fold change from the sham control group.

### 2.8. Quantitative Real-Time Polymerase Chain Reaction

Total RNA was extracted from tissue samples by the TRIzol reagent (Invitrogen, Carlsbad, CA). Then, 2 *μ*g of total RNA was reverse transcribed using the SuperScript RT kit from Invitrogen (Invitrogen, Carlsbad, CA). Quantitative RT-PCR was performed using the ABI PRISM7900 Sequence Detection System (Applied Biosystems) with SYBR Green Master Mix. The sequence-specific oligonucleotide primers (forward and reverse, resp.) were used as follows: NF-*κ*B 5′-GTATGGCTTCCCGCACTATGG-3′; and 5′-TCGTCACTCTTGGCACAATCTC-3′; TNF-*α*: 5′-GTCGTAGCAAACCACCAAGC-3′ and 5′-CTCCTGGTATGAAATGGCAAA-3′; ICAM-1: 5′-GGAGACTAACTGGATGAAAGACGAA-3′ and 5′-TGGCGGCTCAGTGTCTCATT-3′; MCP-1: 5′-GTGTCCCAAAGAAGCTGTAGTATTT-3′ and 5′-GTGCTGAAGTCCTTAGGGTTGA-3′; GAPDH: 5′-GGAAAGCTGTGGCGTGAT-3′ and 5′-AAGGTGGAAGAATGGGAGTT-3′. In order to confirm amplification specificity, the PCR products from each primer pair were subjected to a melting curve analysis and subsequent agarose gel electrophoresis. A control without cDNA was run in parallel with each assay. Each reaction was amplified in triplicate, and ratio results were calculated based on the 2^−ΔΔCT^ method as described previously [26]. Relative mRNA levels were normalized to those of GAPDH and described as the fold change from the sham control group.

### 2.9. Statistical Analysis

Statistics were conducted by SPSS 13.0 software. All data were expressed as mean ± standard deviation (SD). The significance of differences among experimental groups was determined by ANOVA analysis. When a significant difference was detected, the data were further analyzed by Dunnett's multiple range test. A value of *P* < 0.05 was considered statistically significant.

## 3. Results

### 3.1. AS-IV Significantly Ameliorated Renal Dysfunction and Histological Damage in Rats with Ischemic AKI

**Figure 1:**
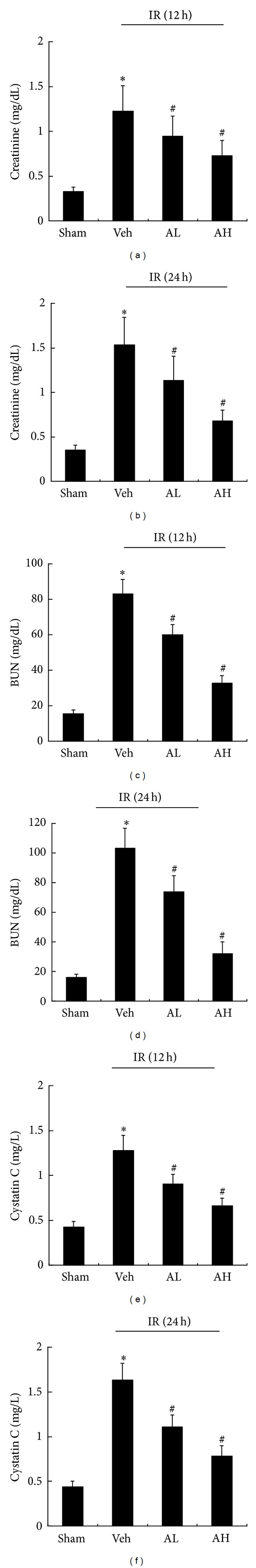
Astragaloside IV(AS-IV) improved renal dysfunction in rats with ischemic AKI. Serum creatinine (a), BUN (c), and cystatin C (e) in sham, vehicle-, or AS-IV-pretreated rats at 12 h of reperfusion. Serum creatinine (b), BUN (d), and cystatin C (f) in sham, vehicle-, or AS-IV-pretreated rats at 24 h of reperfusion. Sham, sham-operated rats treated with normal saline; Veh, ischemia-reperfusion (IR) rats pretreated with carboxymethyl cellulose vehicle alone served as control; AL, IR rats pretreated with AS-IV (10 mg·kg^−1^·d^−1^); AH, IR rats pretreated with AS-IV (20 mg·kg^−1^·d^−1^). Results are expressed as mean ± SD (*n* = 8). *P < 0.05 versus Sham group; ^#^ *P* < 0.05 versus Veh group.

Serum creatinine and BUN levels were significantly elevated in vehicle-pretreated rats compared with sham-operated animals; however, AS-IV pretreatment significantly decreased serum creatinine and BUN levels at 12 h (Figures 1(a) and 1(c)) and 24 h (Figures 1(b) and 1(d)) of reperfusion, respectively. Moreover, rats subjected to renal ischemia exhibited significant increases in serum cystatin C levels compared with sham-operated animals. However, the rats pretreated with AS-IV had significantly lower levels of serum cystatin C at 12 h (Figure 1(e)) and 24 h (Figure 1(f)) of reperfusion, respectively. Therefore, AS-IV pretreatment decreased serum cystatin C levels in rats with ischemic AKI. 

**Figure 2:**
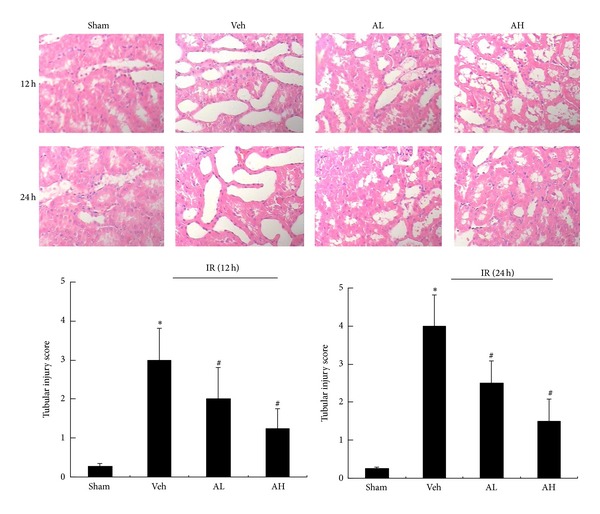
AS-IV significantly ameliorated histological damage in rats with ischemic AKI. Representative hematoxylin and eosin (HE-) stained kidney sections from sham, vehicle-, or AS-IV-pretreated rats at 12 h and 24 h of reperfusion. Histopathologic scoring of tubular injury in kidneys at 12 h and 24 h of reperfusion. Results are expressed as mean ± SD. *P < 0.05 versus Sham group; ^#^ *P* < 0.05 versus Veh group.

Histopathologic scoring indicated that the kidneys from the vehicle-pretreated group had severe tubular damage, as evidenced by tubular necrosis and tubular cell detachment at 12 h and 24 h of reperfusion, respectively (Figure 2). In contrast, AS-IV pretreatment induced significantly less tubular damage in rats with ischemic AKI at the same time point (Figure 2). Quantification of the tubular damage showed markedly diminished histologic features of renal tubular injuries from the kidneys of rats pretreated with AS-IV (Figure 2). Taken together, these findings demonstrated that AS-IV prevented renal dysfunction and histologic damage in ischemic AKI. 

### 3.2. AS-IV Decreased Renal MPO Activity and Inhibited the Activity and mRNA Expression of NF-*κ*B in Rats with Ischemic AKI

**Figure 3:**
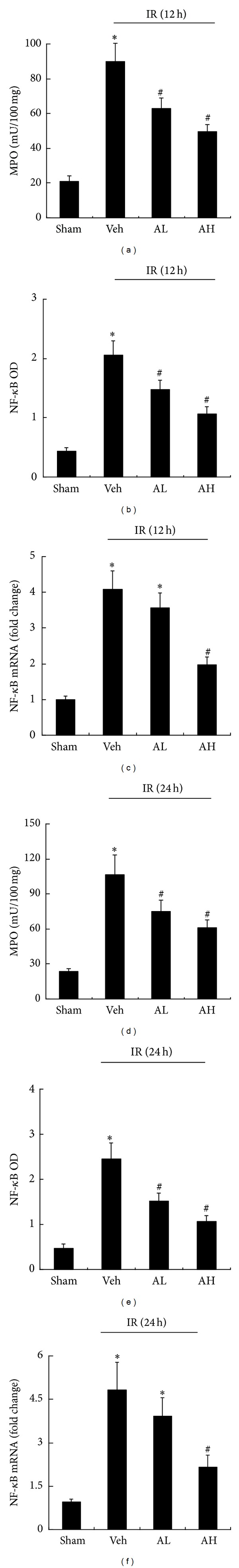
AS-IV decreased renal MPO activity and inhibited the activity and mRNA expression of NF-*κ*B in rats with ischemic AKI. Renal MPO activity (a), NF-*κ*B activity (b), and mRNA expression (c) in sham, vehicle-, or AS-IV-pretreated rats at 12 h of reperfusion. Renal MPO activity (d), NF-*κ*B activity (e), and mRNA expression (f) in sham, vehicle-, or AS-IV-pretreated rats at 24 h of reperfusion. Results are expressed as mean ± SD. *P < 0.05 versus Sham group; ^#^ *P* < 0.05 versus Veh group.

MPO activity, as an indicator of neutrophil infiltration and accumulation in tissues, was detected. Compared with corresponding vehicle-pretreated group, AS-IV pretreatment induced a significant decrease in the renal MPO and NF-*κ*B activity at 12 h (Figures 3(a) and 3(b)) and 24 h (Figures 3(d) and 3(e)) of reperfusion, respectively. Moreover, AS-IV downregulated the mRNA expression of NF-*κ*B in rats with ischemic AKI at 12 h (Figure 3(c)) and 24 h (Figure 3(f)) of reperfusion. The above results demonstrated that AS-IV inhibited AKI-mediated increase in MPO activity, as well as activation and overexpression of NF-*κ*B.

### 3.3. AS-IV Suppressed the Phosphorylation of p65 Subunit of NF-*κ*B in Rats with Ischemic AKI

**Figure 4:**
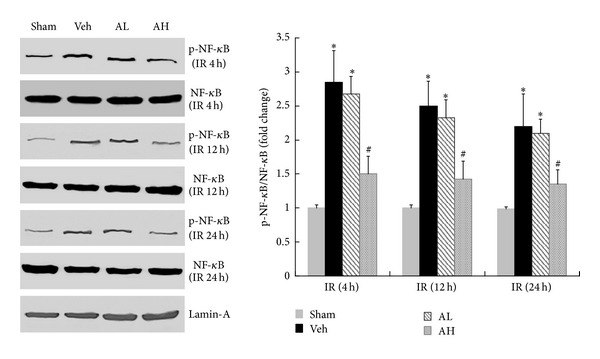
AS-IV suppressed the phosphorylation of p65 subunit of NF-*κ*B in rats with ischemic AKI. Representative Western blot of phosphorylated and total NF-*κ*B in sham, vehicle-, or AS-IV-pretreated rats at 4 h, 12 h, and 24 h of reperfusion. Lamin A was used as a loading control. Results are expressed as mean ± SD. *P < 0.05 versus Sham group; ^#^ *P* < 0.05 versus Veh group.

Compared with the corresponding sham-operated group, there was a significant increase in the phosphorylation of p65 subunit of NF-*κ*B detected by western blotting in vehicle-pretreated group. However, AS-IV pretreatment downregulated the protein expression of the phosphorylated p65 subunit of NF-*κ*B in rats with ischemic AKI at 4 h, 12 h, and 24 h of reperfusion, respectively (Figure 4). Thus, the preventive effects of AS-IV were associated with inhibition of NF-*κ*B activation.

### 3.4. AS-IV Decreased the Serum Levels of MCP-1, ICAM-1, and TNF-*α* in Rats with Ischemic AKI

**Figure 5:**
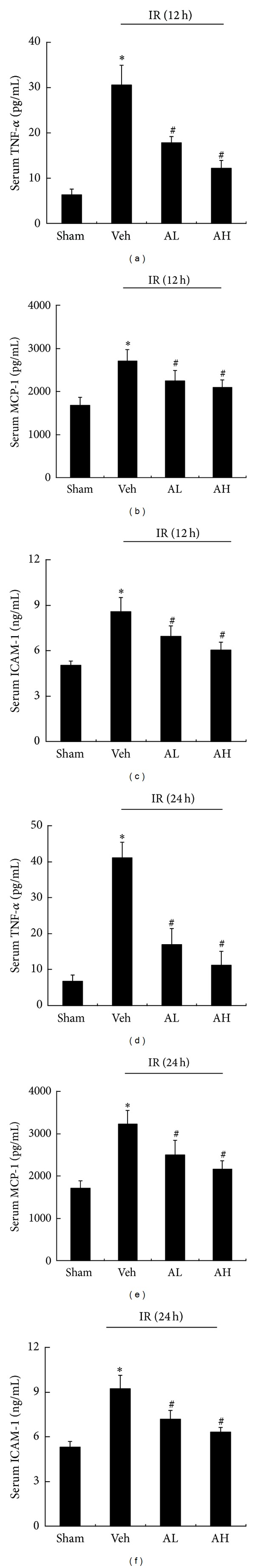
AS-IV decreased the serum levels of TNF-*α*, MCP-1, and ICAM-1 in rats with ischemic AKI. Serum levels of TNF-*α* (a), MCP-1 (b), and ICAM-1 (c) in sham, vehicle-, or AS-IV-pretreated rats at 12 h of reperfusion. Serum levels of TNF-*α* (d), MCP-1 (e), and ICAM-1 (f) in sham, vehicle-, or AS-IV-pretreated rats at 24 h of reperfusion. Results are expressed as mean ± SD (*n* = 8). *P < 0.05 versus Sham group; ^#^ *P* < 0.05 versus Veh group.

Compared with the corresponding sham-operated rats, there was a significant increase in serum levels of MCP-1, ICAM-1, and TNF-*α* at 12 h of reperfusion in rats with ischemic AKI. Pretreatment with AS-IV decreased the serum levels TNF-*α* (Figure 5(a)), MCP-1 (Figure 5(b)), and ICAM-1 (Figure 5(c)) at 12 h of reperfusion in ischemic rats when compared with the vehicle-pretreated rats. These effects were dose-dependent, which were evident at a dose as low as 10 mg/kg and reached the maximal effect at 20 mg/kg of AS-IV. Likewise, the serum levels of TNF-*α* (Figure 5(d)), MCP-1 (Figure 5(e)), and ICAM-1 (Figure 5(f)) were significantly elevated at 24 h of reperfusion in rats with ischemic AKI. All of these abnormalities were prevented by AS-IV. Thus, AS-IV pretreatment significantly reduced the serum levels of MCP-1, ICAM-1, and TNF-*α* in ischemic AKI. 

### 3.5. AS-IV Reduced the Protein Content of MCP-1, ICAM-1, and TNF-*α* in Rats with Ischemic AKI

**Figure 6:**
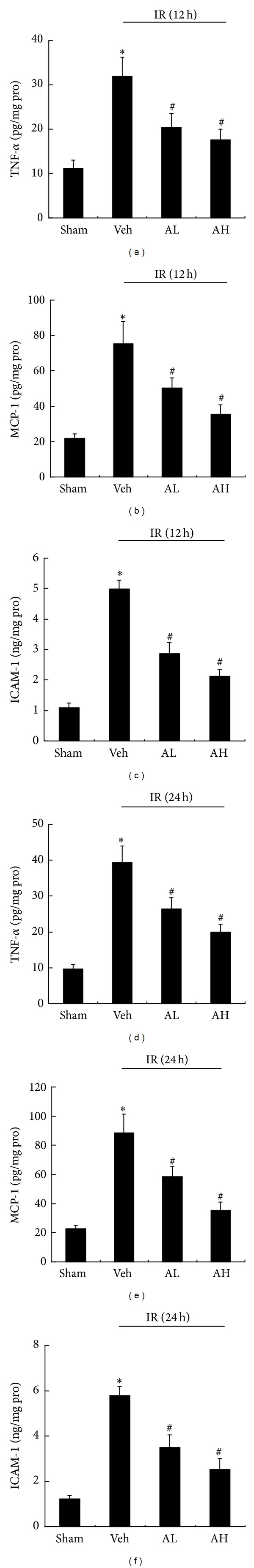
AS-IV reduced the protein content of MCP-1, ICAM-1, and TNF-*α* in rats with ischemic AKI. The protein content of TNF-*α* (a), MCP-1 (b), and ICAM-1 (c) in renal tissues from sham, vehicle-, or AS-IV-pretreated rats at 12 h of reperfusion. The protein content of TNF-*α* (d), MCP-1 (e), and ICAM-1 (f) in renal tissues from sham, vehicle-, or AS-IV-pretreated rats at 24 h of reperfusion. Results are expressed as mean ± SD (*n* = 8). *P < 0.05 versus Sham group; ^#^ *P* < 0.05 versus Veh group.

We observed that the protein content levels of TNF-*α* (Figure 6(a)), MCP-1 (Figure 6(b)), and ICAM-1 (Figure 6(c)) in renal tissues were elevated at 12 h of reperfusion in rats with ischemic AKI, which were partially restored by AS-IV pretreatment. Similarly, the protein content of MCP-1, ICAM-1, and TNF-*α* in the kidney tissues significantly increased at 24 h of reperfusion in rats with AKI when compared with sham-operated rats. However, pretreatment with AS-IV dose-dependently reduced the protein content of TNF-*α* (Figure 6(d)), MCP-1 (Figure 6(e)), and ICAM-1 (Figure 6(f)) at 24 h of reperfusion in rats with ischemic AKI. These results indicated that AS-IV apparently decreased the protein content levels of MCP-1, ICAM-1, and TNF-*α* in kidney tissues from rats with ischemic AKI.

### 3.6. AS-IV Downregulated the mRNA Expression of MCP-1, ICAM-1, and TNF-*α* in Rats with Ischemic AKI

**Figure 7:**
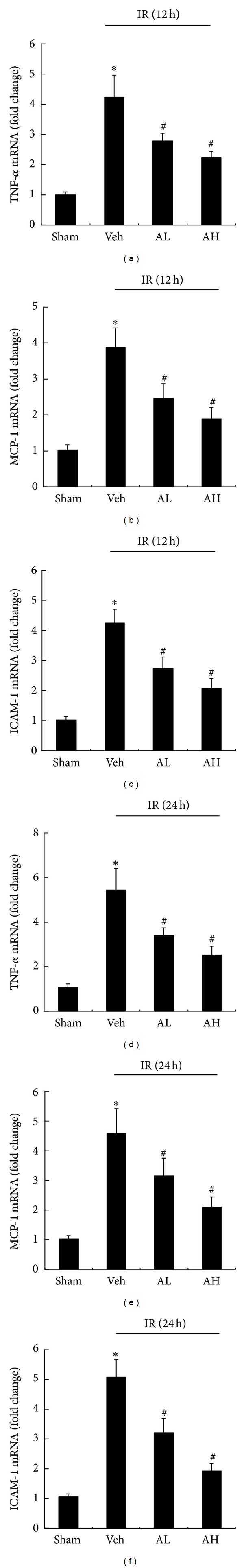
AS-IV downregulated the mRNA expression of TNF-*α*, MCP-1, and ICAM-1 in rats with ischemic AKI. Representative real-time PCR of TNF-*α* (a), MCP-1 (b), and ICAM-1 (c) in renal tissues from sham, vehicle-, or AS-IV-pretreated rats at 12 h of reperfusion. Representative real-time PCR of TNF-*α* (d), MCP-1 (e), and ICAM-1 (f) in renal tissues from sham, vehicle-, or AS-IV-pretreated rats at 24 h of reperfusion. Results are expressed as mean ± SD. *P < 0.05 versus Sham group; ^#^ *P* < 0.05 versus Veh group.

Compared with sham-operated rats, the mRNA expression of inflammatory mediators, MCP-1, ICAM-1, and TNF-*α*, increased significantly at 12 h of reperfusion in rats with ischemic AKI, while pretreatment with AS-IV apparently downregulated the mRNA expression of TNF-*α* (Figure 7(a)), MCP-1 (Figure 7(b)), and ICAM-1 (Figure 7(c)) in rats with ischemic AKI at the same time point. At 24 h of reperfusion, the vehicle-pretreated rats showed enhanced mRNA expression of TNF-*α*, MCP-1, and ICAM-1 while AS-IV pretreatment induced an apparent reduction in the gene expression of TNF-*α* (Figure 7(d)), MCP-1 (Figure 7(e)), and ICAM-1 (Figure 7(f)). All of these effects were dose-dependent, which were evident at a dose as low as 10 mg/kg and reached the peak effect at 20 mg/kg of AS-IV (Figure 7). These results demonstrated that the anti-inflammatory effects of AS-IV were associated with downregulation of inflammatory mediators, such as MCP-1, ICAM-1, and TNF-*α*. 

## 4. Discussion

This study firstly demonstrated that AS-IV, a novel anti-inflammatory agent, ameliorated structural and biochemical abnormalities in a rat model of ischemic AKI through suppressing NF-*κ*B activation and its key downstream inflammatory mediators. Pretreatment with AS-IV apparently reduced inflammatory responses in ischemic rats, as evidenced by a significant decrease in kidney MPO activity and expression of inflammatory mediators. AS-IV pretreatment also significantly suppressed activation and overexpression of NF-*κ*B, as a consequence, the serum and tissue levels of TNF-*α*, MCP-1, and ICAM-1 were further significantly decreased. The preventive effects of AS-IV were further confirmed by the finding that AS-IV pretreatment significantly decreased serum urea, creatinine and cystatin C levels, and tubular damage in ischemic AKI. 

As an attempt to explore the possible mechanisms for the renoprotective effects of AS-IV, we investigated the effects of AS-IV on the NF-*κ*B activity and expression during ischemic AKI. NF-*κ*B, one of the most important transcription factors, regulates expression of the inflammatory genes associated with many pathophysiological conditions, including renal IR injury [16, 27]. Upon stimulation, the inhibitor of NF-*κ*B becomes degraded, and NF-*κ*B releases and translocates into the nucleus where it induces the expression of target genes, most of which encode proteins involved in immune and inflammatory responses [16]. NF-*κ*B undergoes phosphorylation on serine 276 in its p65 subunit and subsequently binds with DNA and promotes the transcription of inflammatory genes, such as cytokines, chemokines, and adhesion molecules [28–32]. Activation of NF-*κ*B has been reported during IR injury, indicating that NF-*κ*B plays a key role in the initiation of inflammatory processes [27]. The crucial role of NF-*κ*B in ischemia/hypoxia induced inflammation is further confirmed by recent studies [33, 34]. The results presented in this study also demonstrated that renal IR injury induced activation of NF-*κ*B, which was consistent with previous study [17]. AS-IV has been reported to have anti-inflammatory activity through inhibiting NF-*κ*B activation and adhesion molecule expression in lipopolysaccharide (LPS-) stimulated HUVECs [22]. However, it is still unknown whether the inhibited effect of AS-IV on inflammatory processes in ischemic AKI is involved in suppression of NF-*κ*B activation. Previous study has reported that phosphorylation on serine 276 is essential for NF-*κ*B p65-dependent cellular responses [35]. Thus, detection of the phosphorylated p65 subunit of NF-*κ*B was effective for evaluating NF-*κ*B activation [16]. Here, the increased expression of phosphorylated p65 subunit of NF-*κ*B was observed in ischemic AKI rats. However, AS-IV pretreatment apparently suppressed NF-*κ*B activation, as evidenced by a decrease in activity and phosphorylation of NF-*κ*B. Previous study demonstrated the activation of NF-*κ*B detected by immunohistochemical staining in a rat model of ischemic AKI [17]. In the present study, we further determined the mRNA expression of NF-*κ*B by quantitative real-time RT-PCR. We found that mRNA expression of NF-*κ*B was increased in renal tissues of ischemic AKI rats, which was significantly suppressed by AS-IV pretreatment. In addition, inhibition of NF-*κ*B by AS-IV also significantly ameliorated renal dysfunction and histological damage in renal IR injury. These results demonstrated that the inhibitory effect of AS-IV on renal inflammation in ischemic AKI rats was partly involved in the suppression of NF-*κ*B activation and overexpression. Moreover, we also tested the effects of AS-IV on NF-*κ*B-dependent luciferase activity and NF-*κ*B p65 mRNA expression in HK-2 cells. Our results showed that AS-IV inhibited NF-*κ*B activity and downregulated the mRNA expression of NF-*κ*B p65 in a dose-dependent manner (data shown in the Supplementary Material available online at http://dx.doi.org/10.1155/2013/284025). Thus, AS-IV may have a direct inhibitory effect on NF-*κ*B in vitro.

We then investigated the effects of AS-IV on the key downstream inflammatory mediators of NF-*κ*B, including TNF-*α*, MCP-1, and ICAM-1. Leukocytes, such as neutrophils and lymphocytes, induce a cascade of proinflammatory responses. Myeloperoxidase (MPO) is critically involved in the induction of organ damage after renal ischemia reperfusion by influencing neutrophil extravasation [36]. Once neutrophils migrate into the ischemic area, they release MPO and cytokines, all of which are involved in tissue injury. In our study, there was a significant increase in MPO activity in ischemic AKI rats, whereas AS-IV pretreatment significantly reduced MPO activity and protected the renal tissue against further injury. Neutrophils are not the only cells that infiltrate after AKI, monocytes/macrophages, DCs, and T cells are also important contributors to ischemic AKI [37]. The effects of AS-IV on these cells need further study. 

Ischemic AKI resulted in the production of the proinflammatory cytokines, such as TNF-*α*, IL-1*β*, and IL-6 [38]. These cytokines initiated neutrophil activation and infiltration and induced not only localized tissue injury but also distant organ injury [39]. The previous study demonstrated that increased MCP-1 expression, mediated by activation of NF-*κ*B, might be responsible for elevated monocyte infiltration during IR injury [27]. In agreement with the above study, we found that the MCP-1 expression was elevated in ischemic AKI. Ischemic AKI also induced expression of a number of adhesion molecules such as ICAM-1, P-selectin, and E-selectin [9, 40]. Application of ICAM-1 antisense oligonucleotides using a topical hydrogel tissue sealant prevented kidney damage in a murine partial nephrectomy/ischemia model [41]. The above studies indicated that many inflammatory mediators, such as cytokines, chemokines, and adhesion molecules were important components of both the initiation and extension of inflammation in ischemic AKI. Thus, the effects of AS-IV pretreatment on the production and expression of inflammatory mediators were further examined in this study. We observed that the levels of TNF-*α*, MCP-1, and ICAM-1 in serum and renal tissues were elevated in rats with ischemic AKI, which were partially restored by AS-IV pretreatment. AS-IV pretreatment also induced an apparent reduction in the gene expression of TNF-*α*, MCP-1, and ICAM-1 in the renal tissue. The reduced TNF-*α*, MCP-1, and ICAM levels in AS-IV-pretreated kidneys were associated with much less MPO activity and less tubular injury. It is no doubt that various cells and inflammatory mediators will be induced by ischemic AKI. The activation of epithelial and possibly endothelial cells during the early initiation phase of ischemic AKI leads to the upregulation of a variety of chemokines and cytokines, such as IL-6, MCP-1, and TNF-*α* [42]. And TNF-*α*, in most cases, frequently induced in the presence of infection, plays a crucial role in the pathogenesis of ischemic AKI. Early upregulation and release of TNF-*α* and NF-*κ*B activation have been observed in renal ischemic injury [43]. In contrast, inhibition of TNF-*α* has been demonstrated to attenuate the decrease in glomerular filtration rate occurring in the renal artery clamp model [44]. These studies demonstrated an important role for TNF-*α* in ischemic AKI. In the present study, AS-IV reduced the protein content and mRNA expression of TNF-*α* in rats with ischemic AKI. Therefore, AS-IV inhibited inflammation and renal histologic damage in ischemic AKI, and the mechanisms might be partly attributed to its ability to inhibit NF-*κ*B activation and its downstream inflammatory mediators, TNF-*α*, MCP-1, and ICAM-1.

We also evaluated the protective effects of AS-IV given after the ischemic insult in rats. Oral administration of AS-IV (10, 20, and 30 mg/kg) at 0 h and 12 h of reperfusion dose-dependently decreased BUN, serum creatinine, and cystatin C levels in IR rats (data shown in the Supplementary Material). Thus, AS-IV administration after the injury protected against ischemic AKI in rats. Moreover, AS-IV did not affect systolic blood pressure in rats (data shown in the Supplementary Material). Therefore, the protective effects of AS-IV on renal function might be not directly associated with the blood pressure and systemic hemodynamic changes. 

Previous study [45] demonstrated that dexamethasone, an anti-inflammatory agent, ameliorated biochemical and histologic AKI after 24 h by a receptor-dependent, nongenomic signaling involving the MEK-ERK 1/2 pathway. Our previous study demonstrated that AS-IV prevented ischemia-induced AKI in rats by inhibiting oxidative stress and apoptosis, and the mechanisms of renoprotection by AS-IV were associated with restoring the balance of Bax and Bcl-2 expression and inhibiting caspase-3 and p38 MAPK activation [46]. Thus, the renoprotective effects of AS-IV were also associated with other effects that could independent of the anti-inflammatory effects, such as antioxidant and antiapoptotic effects. The mechanisms for the renoprotective effects of AS-IV might not be same as that of dexamethasone.

AKI has been recognized as a risk factor for the development of chronic kidney disease (CKD). A recent study [47] characterized an experimental model of CKD induced by ischemic AKI in rats, and the prevention of AKI with spironolactone completely prevented the progression to CKD through inhibiting the activation of fibrotic and inflammatory pathways. In this study, AS-IV, a new anti-inflammatory agent, attenuated biochemical and histologic AKI after 12 h and 24 h of reperfusion in rats. Previous study demonstrated the beneficial effect of AS-IV on the long-term consequences of ischemia reperfusion injury in rat [48]. Thus, AS-IV had renal protective effects on short- and long-term consequences of IR injury in rats. Whether AS-IV administered before or after ischemic AKI protects against CKD needs further study. 

In our previous study, diabetic rats were treated with AS-IV at 5 and 10 mg·kg^−1^·d^−1^, p.o., for 8 weeks, and no significant changes in liver and renal function were observed between AS-IV treated and untreated diabetic rats [49]. Our previous study in STZ-induced diabetic rats further demonstrated that pretreatment with AS-IV at 5 and 10 mg·kg^−1^·d^−1^, p.o., for 14 weeks had no toxic side effects to the liver and renal function [50]. The above results indicated that AS-IV did not cause apparent toxic side effects. However, the clinical trial of AS-IV on ischemic AKI has not been performed. Thus, the adverse effects of AS-IV in human AKI need further investigation. We will try to further investigate the applicability of AS-IV in human ischemic AKI in future studies.

## 5. Conclusion

Taken together, this study demonstrated that pretreatment with AS-IV attenuated structural and biochemical abnormalities in a rat model of ischemic AKI through inhibiting NF-*κ*B mediated inflammatory genes expression. Thus, AS-IV may be a promising treatment for the prevention of ischemic AKI.

## Supplementary Material

To investigate the protective effects of Astragaloside IV (AS-IV) given after the ischemic injury, AS-IV(10, 20 and 30 mg/kg) was orally administered to the rats at 0 h and 12 h of reperfusion in ischemia-induced AKI model. The tail cuff method was used for measuring systolic blood pressure in rats. The systolic blood pressure measurements were performed at the baseline and 12 h or 24 h of reperfusion. AS-IV did not affect systolic blood pressure in rats (data shown in Figure S1). Moreover, AS-IV dose-dependently decreased BUN, serum creatinine and cystatin C levels in AKI rats (data shown in Figure S2 ). Furthermore, we added in vitro experiments to further test the effect of AS-IV on NF-kB in HK-2 cells. Luciferase assay is performed to further confirm the direct inhibitory effect of AS-IV on NF-kB activity in vitro. We also investigated the inhibitory effect of AS-IV on NF-kB p65 mRNA expression in HK-2 cells by Real-time PCR. AS-IV inhibited NF-kB activity and down-regulated the mRNA expression of NF-kB p65 in a dose-dependent manner(data shown in Figure S3 ).
