# Supplementary figures and images for: Preventive Effects of a Natural Anti-Inflammatory Agent, Astragaloside IV, on Ischemic Acute Kidney Injury in Rats
Source: Evid Based Complement Alternat Med. 2013 Jun 19;2013:284025. doi: 10.1155/2013/284025 (PMC3703719; doi:10.1155/2013/284025)

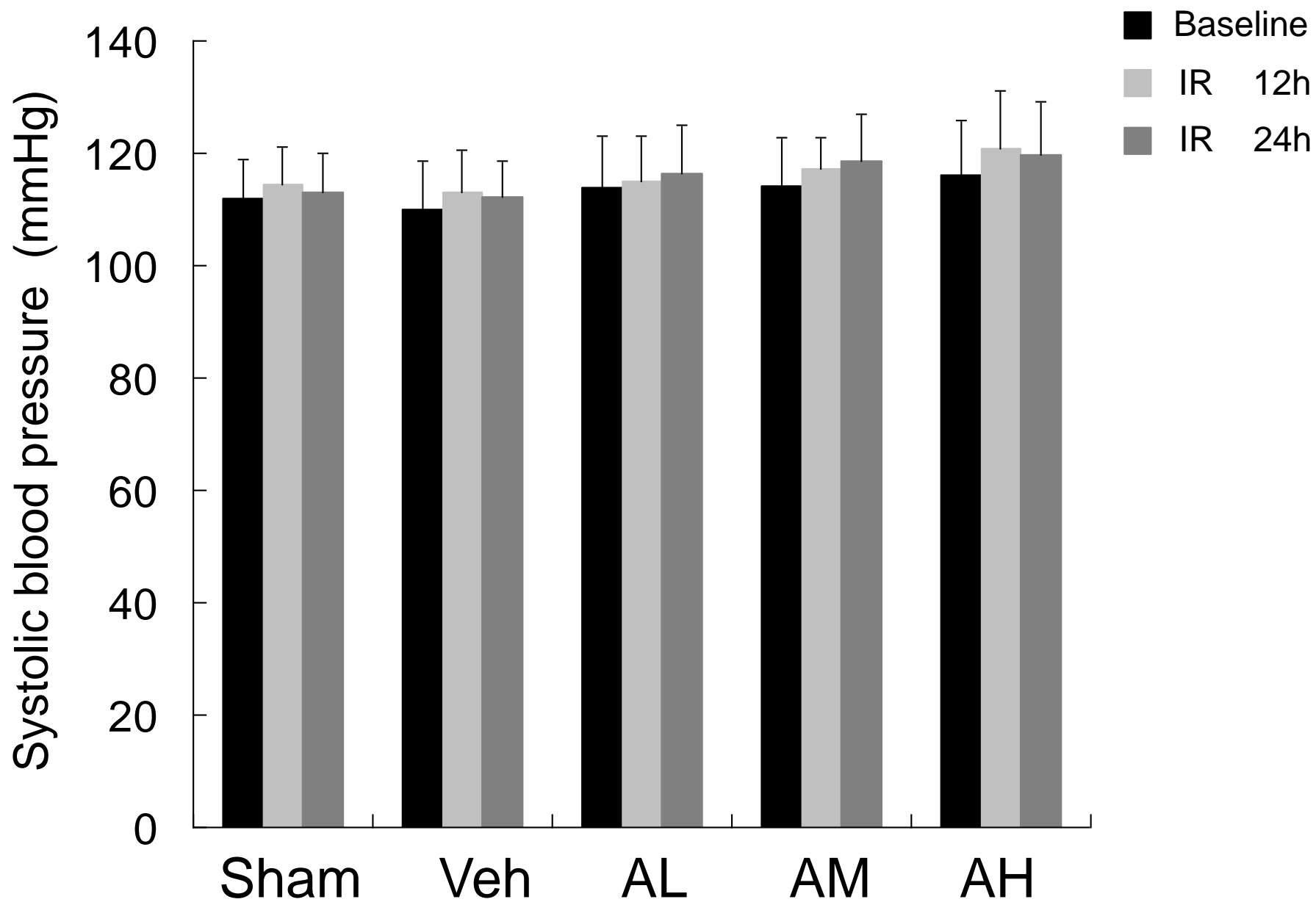

Supplement: Supplementary file 1 — To investigate the protective effects of Astragaloside IV (AS-IV) given after the ischemic injury, AS-IV(10, 20 and 30 mg/kg) was orally administered to the rats at 0 h and 12 h of reperfusion in ischemia-induced AKI model. The tail cuff method was used for measuring systolic blood pressure in rats. The systolic blood pressure measurements were performed at the baseline and 12 h or 24 h of reperfusion. AS-IV did not affect systolic blood pressure in rats (data shown in Figure S1). Moreover, AS-IV dose-dependently decreased BUN, serum creatinine and cystatin C levels in AKI rats (data shown in Figure S2 ). Furthermore, we added in vitro experiments to further test the effect of AS-IV on NF-kB in HK-2 cells. Luciferase assay is performed to further confirm the direct inhibitory effect of AS-IV on NF-kB activity in vitro. We also investigated the inhibitory effect of AS-IV on NF-kB p65 mRNA expression in HK-2 cells by Real-time PCR. AS-IV inhibited NF-kB activity and down-regulated the mRNA expression of NF-kB p65 in a dose-dependent manner(data shown in Figure S3 ). [file 284025.f1.pdf]

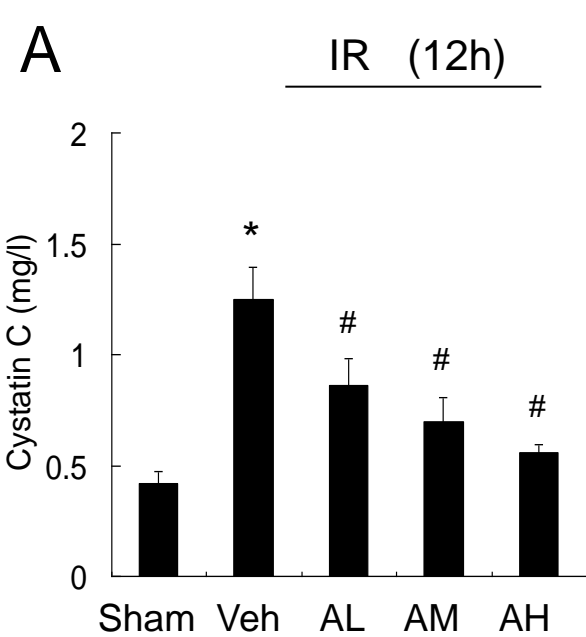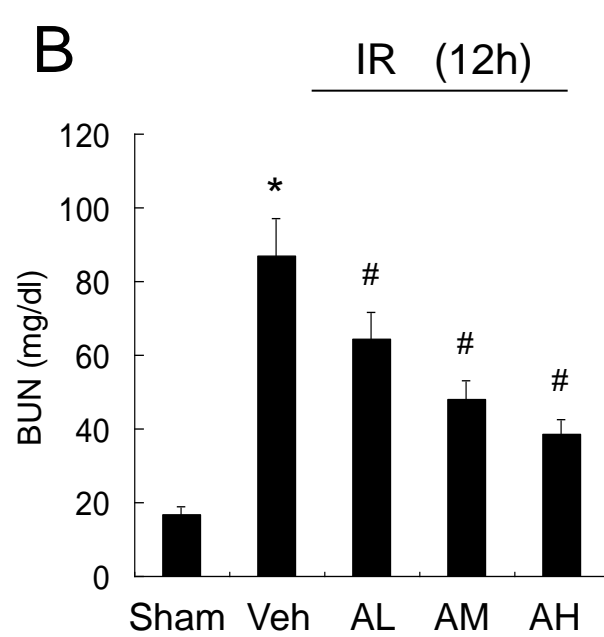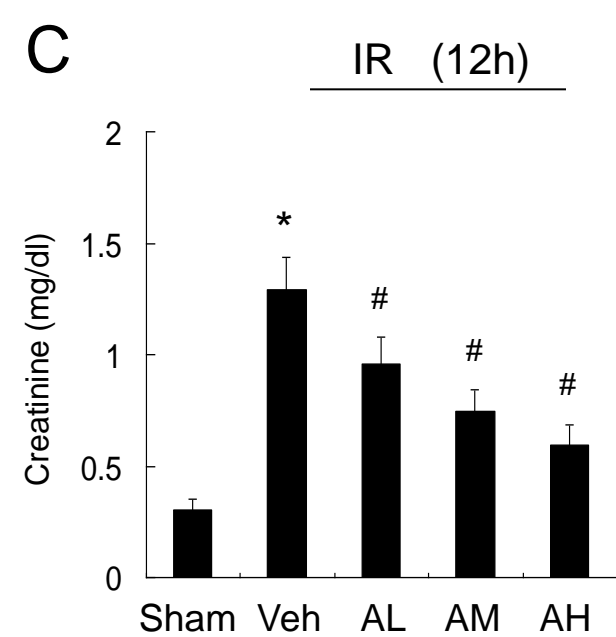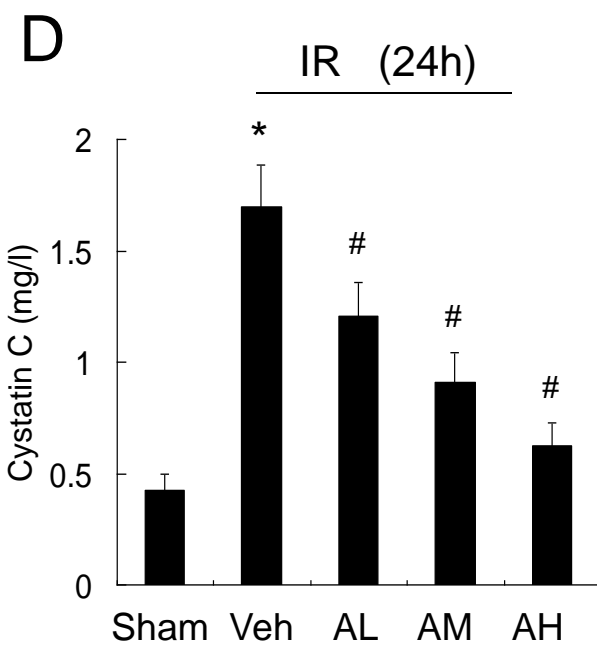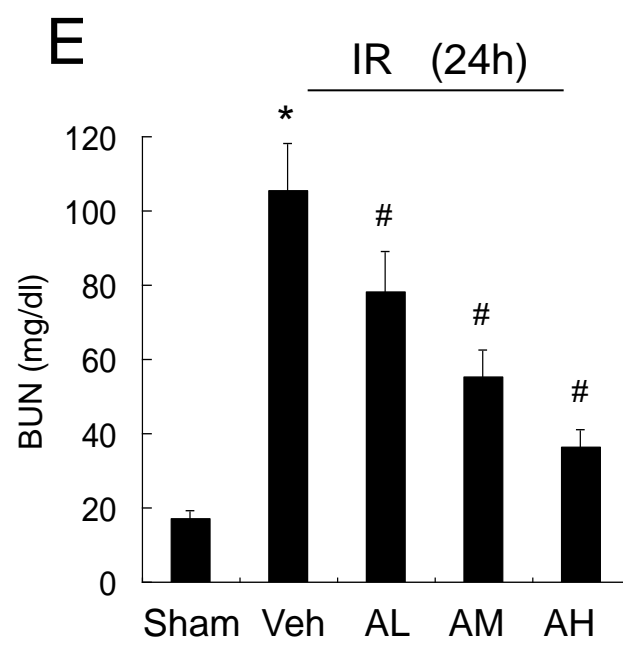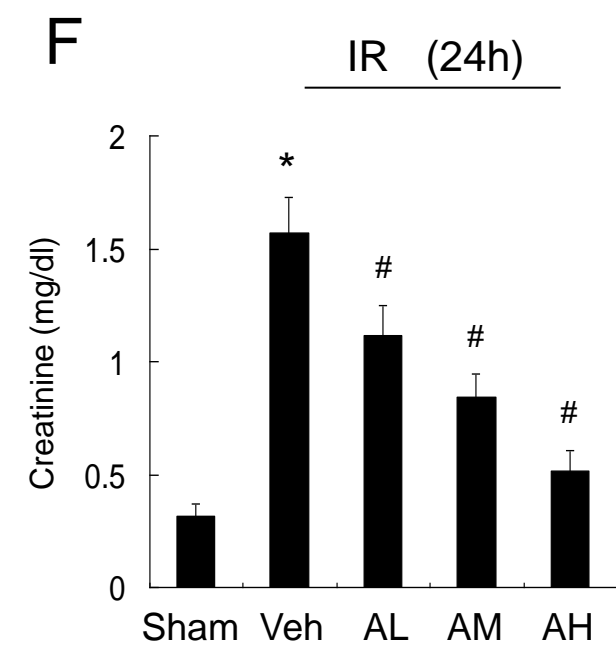

Supplement: Supplementary file 2 [file 284025.f2.pdf]

**A**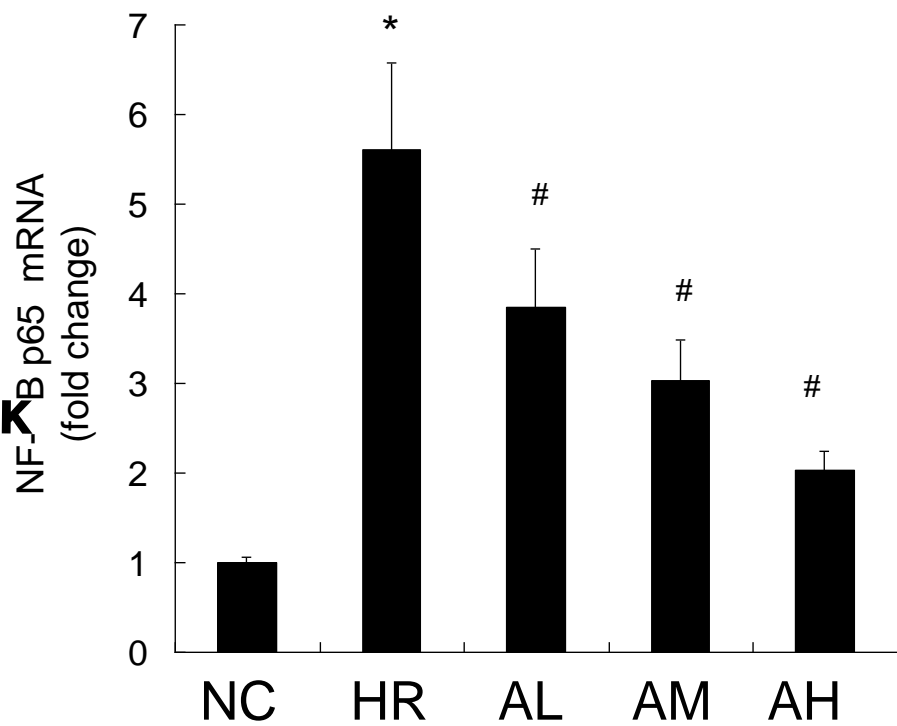**B**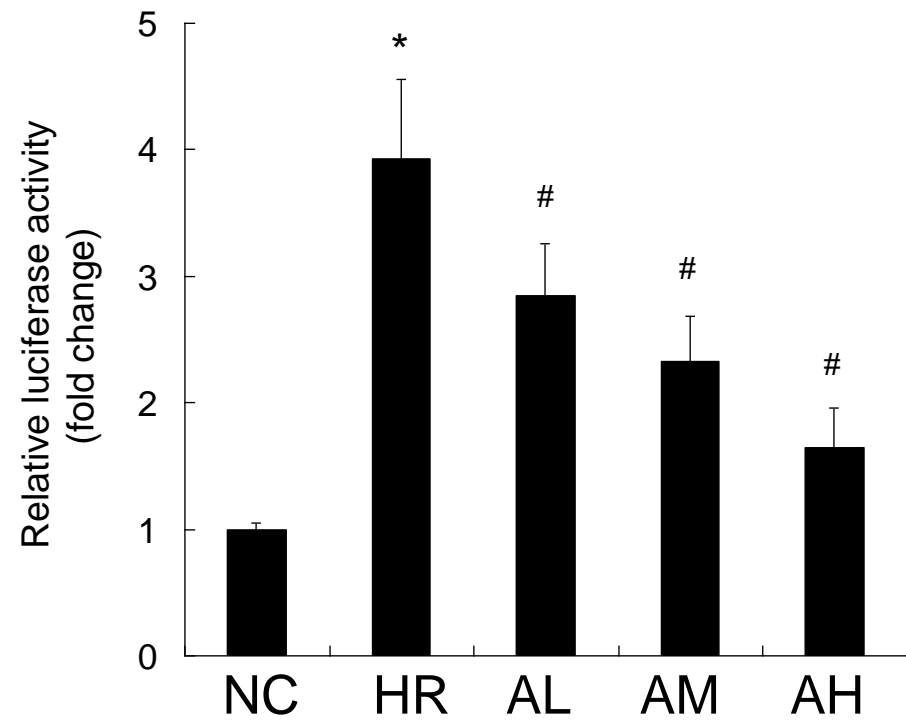

Supplement: Supplementary file 3 [file 284025.f3.pdf]
